# One-Year Monitoring of the Evolution of SARS-CoV-2 Omicron Subvariants Through Wastewater Analysis (Central Italy, August 2023–July 2024)

**DOI:** 10.3390/life15060850

**Published:** 2025-05-24

**Authors:** Alessandra Nappo, Maya Petricciuolo, Giulia Berno, Agnese Carnevali, Cesare Ernesto Maria Gruber, Giulia Bicchieraro, Roberta Spaccapelo, Martina Rueca, Fabrizio Carletti, Pietro Giorgio Spezia, Carolina Veneri, Giuseppina La Rosa, Elisabetta Suffredini, Daniele Focosi, Giovanni Chillemi, Ermanno Federici, Fabrizio Maggi

**Affiliations:** 1 Laboratory of Virology, National Institute for Infectious Diseases Lazzaro Spallanzani—IRCCS, 00149 Rome, Italy; alessandra.nappo@inmi.it (A.N.); cesare.gruber@inmi.it (C.E.M.G.); martina.rueca@inmi.it (M.R.); fabrizio.carletti@inmi.it (F.C.); pietro.spezia@inmi.it (P.G.S.); fabrizio.maggi@inmi.it (F.M.); 2 Bioinformatics Research Unit in Infectious Diseases, National Institute for Infectious Diseases Lazzaro Spallanzani—IRCCS, 00149 Rome, Italy; giovanni.chillemi@inmi.it; 3 Laboratory of Applied and Environmental Microbiology, Department of Chemistry, Biology and Biotechnology, University of Perugia, 06122 Perugia, Italy; maya.petricciuolo@unipg.it (M.P.); agnese.carnevali@dottorandi.unipg.it (A.C.); ermanno.federici@unipg.it (E.F.); 4 Department of Medicine and Surgery and Center of Functional Genomics (C.U.R.Ge.F), University of Perugia, 06132 Perugia, Italy; giulia.bicchieraro@unipg.it (G.B.); roberta.spaccapelo@unipg.it (R.S.); 5 National Center for Water Safety (CeNSia), Istituto Superiore di Sanità, 00161 Rome, Italy; carolina.veneri@guest.iss.it (C.V.); giuseppina.larosa@iss.it (G.L.R.); 6 Department of Food Safety, Nutrition and Veterinary Public Health, Istituto Superiore di Sanità, 00161 Rome, Italy; elisabetta.suffredini@iss.it; 7 North-Western Tuscany Blood Bank, Pisa University Hospital, 56124 Pisa, Italy; daniele.focosi@gmail.com; 8 Department of Experimental Medicine, University of Rome “Tor Vergata”, 00133 Rome, Italy

**Keywords:** SARS-CoV-2, viral variants, wastewater surveillance, next-generation sequencing, RT-qPCR

## Abstract

Wastewater surveillance has proven to be a cost-effective, non-invasive method for monitoring the spread and evolution of SARS-CoV-2, yet its value during today’s low-incidence phase is still being defined. Between August 2023 and July 2024, 42 composite wastewater samples were collected in Perugia, Italy and analyzed using RT-qPCR and whole-genome sequencing to identify circulating SARS-CoV-2 lineages. In parallel, clinical samples (respiratory tract samples) were collected and analyzed, allowing for direct comparisons to confirm the robustness of the wastewater findings. The sewage viral loads ranged from 8.9 × 10^5^ to 4.9 × 10^7^ genome copies inhabitant^−1^ day^−1^, outlining two modest community waves (September–December 2023 and May–July 2024). Sequencing resolved 403 Omicron lineages and revealed three successive subvariant phases: (i) XBB.* dominance (August–October 2023), when late-Omicron XBB subvariants (mainly EG.5.* and XBB.1.5) accounted for almost all genomes; (ii) a BA.2.86/JN surge (November 2023–March 2024), during which the BA.2.86 subvariant, driven mainly by its JN descendants (especially JN.1), rapidly displaced XBB.* and peaked at 89% in February 2024; and (iii) KP.* takeover (April–July 2024), with JN.1-derived KP subvariants rising steadily and KP.3 reaching 81% by July 2024, thereby becoming the dominant lineage. Comparisons of data from wastewater and clinical surveillance demonstrated how the former presented a much higher diversity of circulating viral lineages. Importantly, some subvariants (including BA.2.86*) were detected in wastewater weeks to months prior to clinical identification, and for longer periods. Taken together, the obtained data validated wastewater surveillance as an effective early warning system, especially during periods of low infection prevalence and/or limited molecular testing efforts. This methodology can thus complement clinical surveillance by offering valuable insights into viral dynamics at the community level and enhancing pandemic preparedness.

## 1. Introduction

Monitoring the evolution and spread of viral pathogens, such as SARS-CoV-2, in the population is crucial for preventing and controlling epidemics and ensuring an effective public health response. Despite the WHO having declared the end of the COVID-19 public health emergency of international concern since May 2023, robust surveillance systems are still needed, as the virus continues to evolve, posing significant challenges in term of diagnostic techniques, treatment strategies, and vaccine effectiveness [1]. In this respect, in Italy, the impact of the virus at the national level is continuously monitored through reports produced by the Istituto Superiore di Sanità (ISS), in which SARS-CoV-2 infection cases are reported [2]. Nevertheless, the end of the COVID-19 emergency has exacerbated the biases associated with clinical tests, thereby urging the need for alternative monitoring methods. Wastewater analysis provides a timely overview of virus dissemination, representing pooled genetic information from various types of biological samples, and including a mixture of different viral lineages [3,4,5,6]. This method can intercept viruses from individuals with mild symptoms or who are asymptomatic, who might otherwise escape traditional tracking systems. For human-restricted viruses, wastewater monitoring measures the diversity of the overall viral population at the population level, although it cannot discriminate spread based on demographic factors. It enables the monitoring of viral spread in each geographic area or period in an anonymous and non-invasive way, overcoming potential sampling bias and privacy issues [7,8,9,10,11,12]. Since SARS-CoV-2 can be excreted in the stool and urine of both symptomatic and asymptomatic individuals and is detectable in wastewater, its quantification and subsequent sequencing from these samples is a valuable method to address limitations inherent in clinical-based epidemiological approaches [13,14,15,16]. This method provides an impartial depiction of community-wide infection dynamics and has become a valuable tool to monitor the emergence of SARS-CoV-2 lineages Additionally, with the global decrease in SARS-CoV-2 cases and a significant reduction in molecular testing frequency, wastewater analysis has emerged as an effective complementary tool to support clinical surveillance [17].

Since 2000, the World Health Organization (WHO), in collaboration with regional agencies such as the European Centre for Disease Prevention and Control (ECDC) and the US CDC, has maintained a tiered nomenclature to classify SARS-CoV-2 lineages according to their potential threat to global public health. Lineages are ranked according to their (i) growth advantage and transmissibility, (ii) immune escape capacity, (iii) disease severity, and (iv) impact on diagnostics, vaccines, or therapeutics [18]. Variants of concern (VOCs) are the highest risk category: they show clear evidence of increased transmissibility and either immune escape, clinical severity, or significant diagnostic/therapeutic impact. Historical examples include Alpha (B.1.1.7), Delta (B.1.617.2), and Omicron (B.1.1.529 and its descendants). Below this level, variants of interest (VOIs) show genetic changes of concern or early epidemiological signals, but they lack definitive evidence of additional clinical or public health impact. Lineages that warrant closer monitoring but do not (yet) meet the VOI criteria are tracked as variants under monitoring (VUMs). This dynamic classification, updated regularly, underpins global risk assessment, guides vaccine-strain decisions, and helps authorities allocate testing and sequencing resources efficiently. There are currently no SARS-CoV-2 lineages meeting the VOC criteria, but the ECDC has classified some Omicron subvariants as VOIs and VUMs. The Omicron variant (B.1.1.529) rapidly diversified into multiple sublineages that have played a dominant role in shaping the global epidemiological landscape of COVID-19. Early subvariants such as BA.1 and BA.2 exhibited substantial immune evasion, leading to widespread breakthrough infections [19,20,21]. BA.2, in particular, demonstrated higher transmissibility compared to BA.1 and contributed to sustained waves of infections globally in early 2022 [22]. Subsequent subvariants such as BA.4 and BA.5, which emerged in mid-2022, had further increased immune escape potential and became dominant due to their ability to partially evade neutralizing antibodies [23]. The BQ.1, BQ.1.1, and XBB subvariants, detected later in 2022 and into 2023, introduced additional spike mutations (particularly in the receptor-binding domain) [24]. In 2023, several SARS-CoV-2 XBB descendants, notably EG.5.1, were predominant worldwide, but since July 2023, a new subvariant BA.2.86 (also referred to as “Pirola”) was identified and spread globally, raising attention due to its numerous mutations [25]. With time, genetic differentiation within BA.2.86 led to the emergence of several different sublineages (namely, BA.2.86.1 to BA.2.86.7, and their descending aliases), altogether referred as BA.2.86*. The BA.2.86 subvariant has then rapidly started to evolve, with the emergence of the JN. * subvariant. Furthermore, JN.1 (an alias for BA.2.86.1.1) evolved into the KP.* subvariant (JN.1.11.1.*). The KP.2 subvariant (JN.1.11.1.2) is notable for the spike protein mutations F456L, R346T, and V1104L, which potentially increase the virus’s transmissibility and immune-evasive characteristics. At the time of writing, JN.1, KP.2, KP.3, LB.1, and LP.8 are among the most widespread subvariants worldwide [26].

Wastewater monitoring has proven to be effective in tracking the prevalence of SARS-CoV-2, offering a time advantage for identifying new and emerging lineages before they are detected in clinical specimens [27,28]. This capability enables the early prediction of the epidemic curve by identifying potential new lineages of clinical interest [29]. Wastewater analysis has been used to identify the Omicron variant (B.1.1.529), demonstrating early detection of the variant up to 10–14 days ahead or showing temporal concordance with clinical sample detection [27,30]. Several studies have also reported the identification and characterization in wastewater samples of Omicron subvariants that had not been detected through clinical surveillance [31]. In Italy, wastewater surveillance was used to assess the spread of the Omicron variant, highlighting its increase and progression towards replacing the Delta variant earlier than clinical data indicated [32]. In line with the WHO’s recommendations to remain vigilant, monitor, and report on different Omicron lineages, researchers detected Omicron subvariants, including BQ.1/BQ.1.1 and rare ones such as XBB.1 and BA.2.75, in wastewater before these subvariants were reported in clinical cases [33]. A digital PCR (dPCR) assay for the rapid detection of BA.2.86 and its descendants, including JN.1, in wastewater samples was developed and used to test samples collected in Italy from September 2023 to January 2024, detecting the presence of BA.2.86 as early as October 2023 [34].

While numerous studies have described the characterization of SARS-CoV-2 lineages from wastewater, only a few studies have focused on BA.2.86 subvariants in wastewater [35,36,37,38,39]. Accordingly, we designed a one-year wastewater-based epidemiological campaign in the city of Perugia (Central Italy), with three specific objectives: First, to quantify community-level SARS-CoV-2 circulation by measuring viral loads in weekly composite influent samples collected from August 2023 to July 2024. Second, to map the temporal turnover of all circulating lineages by whole-genome sequencing of the same samples. Third, to compare wastewater-derived lineage profiles with concurrent clinical sequencing to assess the lead time and added value of wastewater surveillance during a period of low testing intensity. Because there are currently no SARS-CoV-2 lineages classified as VOCs, we focused our attention on BA.2.86 subvariants classified as VOIs according to the ECDC [40].

## 2. Materials and Methods

### 2.1. Samples Collection

A total of 42 wastewater samples (50 mL) were collected as a part of the national SARS-CoV-2 surveillance program in wastewater (SARI project, EU Commission Recommendation 2021/472) between August 2023 and July 2024. Sampling was conducted monthly until October (when BA.2.86.* was first detected), and weekly thereafter. Samples were taken at the inlet of the wastewater treatment plant (WWTP) of Pian della Genna, Perugia (Umbria, Italy), which has a designed capacity of 90,000 population equivalent (PE). Sewage was collected as 24h composite samples, and the daily flow rate (m^3^ d^−1^) was recorded for data normalization. Samples were stored at 4 °C and analyzed within 48h.

### 2.2. Sample Processing and RNA Extraction

Wastewater samples were processed according to the standardized national protocol [41]. Briefly, 45 mL of sample was heat-inactivated at 56 °C for 30 min for safety reasons and then centrifuged at 4500× *g* for 30 min at 4 °C to eliminate debris. Then, 40 mL of supernatant, supplemented with polyethylene glycol 8000 (8% *w*/*v*) and NaCl (0.3 M), was centrifuged at 12,000× *g* for 120 min at 4 °C to concentrate the viral particles. The pellet was lysed with 2 mL of NucliSENS lysis buffer, mixed with NucliSENS easyMAG Magnetic Silica, and the RNA was extracted using the eGENE-UP system (bioMerieux, Marcy-l’Étoile, France), following the manufacturer’s instructions. RNA was eluted in 100 μL of TE, purified with the OneStep PCR Inhibitor Removal Kit (Zymo Research, Irvine, CA, USA), and supplemented with 1 U/μL of RiboLock RNase Inhibitor (Thermo Fisher Scientific, Waltham, MA, USA) before storing it at −80 °C. To monitor concentrations and extraction efficiency, 100 μL of control murine norovirus (MNV) supplied by the Italian Istituto Superiore di Sanità (ISS) was spiked in all samples before the first centrifugation.

### 2.3. Detection and Quantification of SARS-CoV-2

RT-qPCR targeting the ORF1b region (nsp14) of the viral genome of SARS-CoV-2, along with data analysis, was performed as described in the national protocol [41]. Accordingly, samples were classified as positive if the cycle threshold (Ct) was lower than 40 (Ct < 40). The standard curve for the quantification of the genomic copies (g.c.)/μL of the targeted SARS-CoV-2 genomic region was prepared using serial dilutions of a standard dsDNA supplied by the ISS. All of the reactions were carried out in technical duplicate, and in all runs, quality controls were included (negative control, positive control, inhibition control, process efficiency control, and RT-control). Quantitative data normalization was carried out as indicated by the national protocol [41], as follows:

SARS-CoV-2 g.c. inh^−1^ d^−1^ = (SARS-CoV-2 g.c. mL ^−1^ × daily flow rate m^3^ d^−1^)/PE

### 2.4. Sequencing of SARS-CoV-2 Variants in Wastewater Samples

Amplicon next-generation sequencing of SARS-CoV-2 was performed by preparing sequencing libraries using the Ion AmpliSeq^®^ SARS-CoV-2 Insight Research Assay (ThermoFisher, Waltham, MA, USA), following the manufacturer’s instructions.. Sequence fragments partially overlapping and covering the whole SARS-CoV-2 genome sequence were obtained. Next-generation sequencing (NGS) was finally carried out on the Ion Torrent Gene Studio S5 Prime (GSS5 Prime) platform using the Ion 520 or 530™ Chip (ThermoFisher, Waltham, MA, USA) to obtain 5.0 × 10^5^ reads per sample.

### 2.5. Bioinformatics Analysis of SARS-CoV-2 Variants in Wastewater Samples

Ion Torrent raw data bam files were converted to FASTQ format using samtools software v.1.13 [42] and filtered by quality checking using Trimmomatic v.0.39 [43]. Quality-filtered raw read sequences were aligned to the reference genome of SARS-CoV-2 (Wuhan-Hu1/2019, MN908947; RefSeq ID NC_045512.2) with the Burrows–Wheeler Alignment (BWA) MEM software v.0.7.12 [44]. All reads that did not map to the reference genome were discarded, and the read-mapping file was converted into “sorted-bam” using samtools software v.1.13 [42]. To remove primer sequences from the aligned SARS-CoV-2 reads, the tool iVar v.1.4.3 was employed for accurate trimming based on the amplicon sequencing protocol using - m 30 - k -e -s 4 - q 20 parameters [45]. The primer sequences were defined in a BED file, which included the coordinates of the primers used during PCR amplification. To detect SARS-CoV-2 lineages from the sequencing data, the Freyja tool was employed for variant calling and to clarify mixed viral populations [46]. To represent every SARS-CoV-2 lineage in the global phylogeny, the Freyja tool implements a continuously up-to-date barcode library of lineage-defining mutations. The SARS-CoV-2 Wuhan-Hu1/2019 reference genome was used to ensure accurate alignment and variant identification. Freyja’s process was used to estimate the relative abundance of different SARS-CoV-2 lineages in wastewater samples. The results provided a detailed profile of the circulating variants within the wastewater samples, helping to identify the predominant and emerging lineages. Variant calls were aggregated, and subsequent analyses were conducted using R v.4.2.1 and RStudio v.4.2.1. The specific SARS-CoV-2 lineages predicted by Freyja were summarized into major lineages, according to the Pango nomenclature [47]. All analyses were performed on the GALILEO100 supercomputer of CINECA, consisting of several nodes, each with 2 x Intel CascadeLake 8260 CPUs (Intel, Santa Clara, CA, USA), with 24 cores each, 2.4 GHz, and 384GB RAM. FASTQ files were uploaded to the NCBI Sequence Read Archive (SRA) (accession number PRJNA1250441).

### 2.6. Phylogenetic Analysis

Three wastewater samples, representative of SARS-CoV-2’s genomic evolution during the period under investigation, were also selected for phylogenetic analysis. For these samples, raw reads with a mean quality Phred score >20 were trimmed using Trimmomatic software v.0.39 [43]. Whole SARS-CoV-2 genomes were assembled using the Easy-to-Use SARS-CoV-2 Assembler pipeline (ESCA): a novel reference-based genome assembly pipeline specifically designed for SARS-CoV-2 data analysis [48]. The assembled genomes were also controlled using Geneious Prime v.2019.2.3. For phylogenetic analysis, full-length SARS-CoV-2 sequences available on the same period of selected samples (August 2023, January 2024, and May 2024) were retrieved from GISAID [49] and analyzed as follows. All European sequences with high coverage classified as environmental samples were considered in the analysis. Multi-sequence alignment was performed with MAFFT v7.271 [50], whole-genome alignment was manually controlled, and 5′ and 3′ UTRs were excluded from further analysis. Maximum likelihood (ML) phylogenetic analysis was performed with IQ-TREE v.1.6.12 [51]; the best tree model was selected using ModelFinder [52]; the best trees were found by performing 5000 ultrafast bootstrap replicates. The strain Whuan-Hu-1 was used as a phylogenetic outgroup (EPI_ISL_402124). Pango lineages of consensus sequences were obtained with Nextclade [53].

### 2.7. Assessment of SARS-CoV-2 Variants in Clinical Samples

A total of 236 respiratory tract samples collected between August 2023 and July 2024 from the hospital and local medical center located in Perugia, Italy (Azienda Ospedaliera di Perugia, and Unità Sanitaria Locale (USL) Umbria 1), and which tested positive by RT-qPCR for the presence of the SARS-CoV-2 genome, were processed as previously described [54]. Briefly, the samples were concentrated by overnight precipitation at −80 °C with 0.1% ammonium acetate (5 M). Then, viral RNA was extracted using the Seegene STARlet IVD automated extraction platform together with the STARMag 96 X 4 Viral DNA/RNA 200 C Kit (Seegene, Seoul, Republic of Korea), following the manufacturer’s instructions. Libraries were prepared using the COVIDSeq assay kit (Illumina, San Diego, CA, USA), based on reverse transcription of the whole viral genome, followed by the amplification of 98 viral target regions using the ARTIC V4.1 primer pool. Sequencing was performed with the Illumina MiSeq platform by using a reagent kit (Illumina) with the 2 × 150 paired-end method. Then, the analysis of the FASTQ files was performed using the DRAGEN (Dynamics Read Analysis for GENomics) pipeline’s COVID Lineage app v4.0.3 for read alignment to the SARS-CoV-2 reference genome (Wuhan-Hu1/2019) and assignment of sequence variants.

## 3. Results

### 3.1. Estimation of Circulating SARS-CoV-2 Quantities

**Figure 1:**
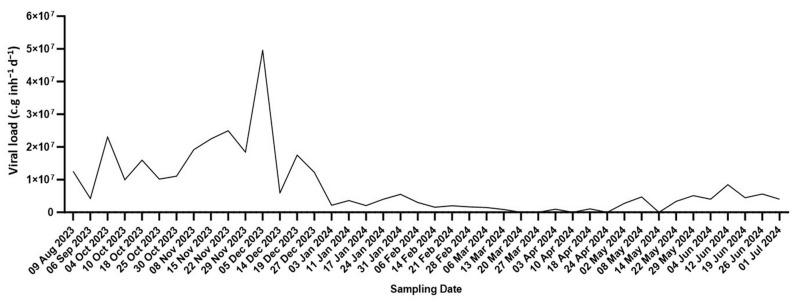
Quantitative trend analysis of SARS-CoV-2 viral load as determined by wastewater analysis: Temporal trends of SARS-CoV-2 viral load (expressed as g.c. inh^−1^ d^−1^) were measured by RT-qPCR in RNA samples extracted from urban wastewater collected between 9 August 2023 and 1 July 2024 from the wastewater treatment plant of Pian della Genna (Perugia, Italy).

From August 2023 to July 2024, 42 wastewater samples were collected from the wastewater treatment plant (WWTP) of Pian della Genna (Perugia, Italy) for the national SARS-CoV-2 surveillance program and analyzed by RT-qPCR. As reported in Figure 1, the viral loads ranged from 8.9 × 10^5^ to 4.9 × 10^7^ c.g. inh^−1^ d^−1^, peaking between September and December 2023, with a smaller rise observed from May to July 2024.

### 3.2. Overview of SARS-CoV-2 Circulating Lineages

**Figure 2:**
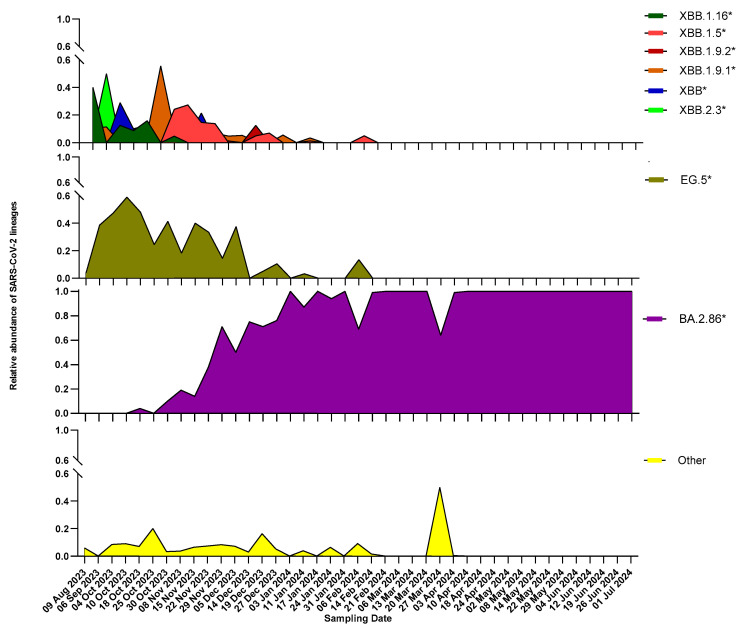
Relative abundances of the main SARS-CoV-2 lineages circulating in the studied area, as assessed by wastewater analysis: SARS-CoV-2 lineages in wastewater samples collected between 9 August 2023 and 1 July 2024 from the wastewater treatment plant of Pian della Genna (Perugia, Italy) were determined using amplicon next-generation sequencing. Lineage identification and calculation of relative abundances at each timepoint were carried out using Freyja’s tool. For clarity, the main lineages found are shown in different panels, with the first and second depicting a detailed division of Omicron XBB lineages, the third grouping all Omicron BA.2.86 lineages together (to highlight their appearance and the gradual replacement of XBB* lineages), and the fourth reporting all other lineages that were not classified in the previous two groups.

We investigated the SARS-CoV-2 lineages in all wastewater samples collected between August 2023 and July 2024, using amplicon next-generation sequencing. By using Freyja’s tool (v1.5.1), we were able to identify and assign consensus lineages for 41 samples (97.6% of the total). As expected, the wastewater samples were characterized by a miscellaneous collection of viral variants, ranging from 2 to 200 in a single sample, and allowed for the overall detection of 403 different sublineages. Figure 2 shows the occurrence of the main SARS-CoV-2 lineages over time. In August 2023, Omicron XBB subvariants such as XBB.1.16* were prominent, with XBB.1.5*, XBB.1.9*, XBB.*, and XBB.2.3* less common. By October to November 2023, the prevalence of these XBB subvariants (XBB.1.5*, XBB.2.3*, XBB.1.16*, and XBB.1.9.1*) decreased, while EG.5* became more common. Despite its decline, XBB.1.5* remained relatively abundant until December 2023 and was present until the beginning of February 2024. An important change was observed starting in mid-October 2023, with the first detection of the BA.2.86 subvariant. Thereafter, the prevalence of BA.2.86 and its descendants rapidly increased, indicating a growing circulation within the general population, and it became exclusively present from April 2024 until the end of the study.

### 3.3. Characterization of BA.2.86 and Its Descendants

**Figure 3:**
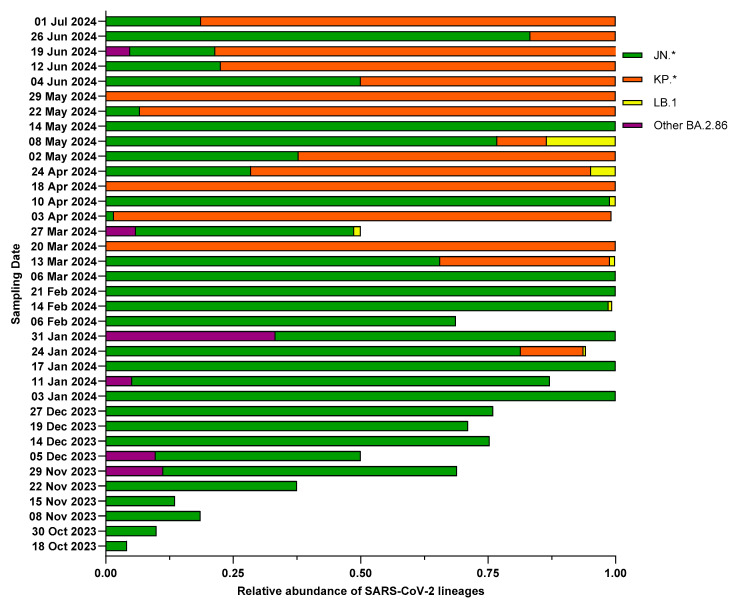
Relative abundances of the main circulating BA.2.86 lineages, as assessed by wastewater analysis: Wastewater samples collected between 18 October 2023 (date of first appearance of Omicron BA.2.86) and 1 July 2024 from the wastewater treatment plant of Pian della Genna (Perugia, Italy) were analyzed by amplicon next-generation sequencing, and lineages were identified with Freyja’s tool. The relative abundances of the main BA.2.86 lineages (grouped into JN.*, KP.*, LB.1, and other BA.2.86) are shown only for wastewater samples showing the presence of Omicron BA.2.86*.

Considering the emergence of BA.2.86 from October 2023 and its high prevalence in wastewater during the period of this study, we focused on BA.2.86 descendants (i.e., JN.*, KP.*, LB.1, and other BA.2.86*) in order to understand their temporal trends (Figure 3). From October 2023 until the first half of January 2024, only JN.* and other BA.2.86 lineages were found. On 24 January 2024, KP.* and LB.1 lineages were identified for the first time, at 12.3% and 0.5% prevalence, respectively. By the second half of March 2024, KP.* were generally more prevalent than other lineages, while LB.1 was sporadically found, albeit with increasing abundance and only until 8 May 2024.

**Figure 4:**
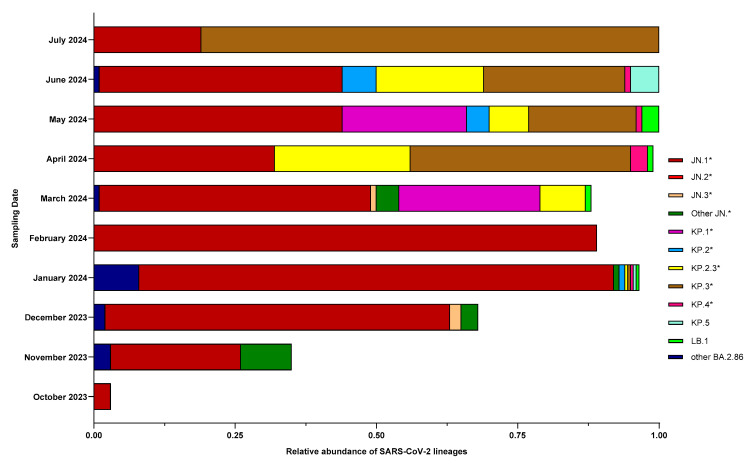
Monthly characterization of circulating BA.2.86 lineages, as assessed by wastewater analysis: Data obtained by amplicon next-generation sequencing and Freyja analyses of wastewater samples collected between 18 October 2023 (date of first appearance of Omicron BA.2.86) and 1 July 2024 from the wastewater treatment plant of Pian della Genna (Perugia, Italy) are reported, aggregated by month, averaging the relative abundances of the main BA.2.86 lineages detected, namely, JN.1, JN.2, JN.3, KP.1, KP.2, KP.2.3, KP.3, KP.4, KP.5, and LB.1 (lineages JN.4 to JN.19 and all other BA.2.86 lineages are grouped into Other JN.* and *Other BA.2.86*, respectively).

After characterizing the abundance of the main circulating BA.2.86 lineages, we conducted a more detailed analysis by month, segregating BA.2.86* into JN.1, JN.2, JN.3, KP.1, KP.2, KP.2.3, KP.3, KP.4, KP.5, and LB.1, for which the monthly averages are shown in Figure 4. All lineages identified after JN.3 (JN.4-JN.19) were categorized as “Other JN.*”, while BA.2.86 lineages that were not JN.*, KP.*, and LB.1 were classified as “Other BA.2.86”. The results showed that the JN.1*, after being detected in October 2023, increased in the following months, becoming predominant in January and February 2024, with an average of 84% and 89%, respectively. Starting from March 2024, when the average abundance of JN.1* decreased to 48%, other lineages appeared: KP.1*, KP.2.3*, and LB.1, accounting for 25%, 8%, and 1%, respectively. By April 2024, there was a further reduction in JN.1* to 32%, along with an increase in KP.2.3* to 24%, KP.3* at 39%, and KP.4* at 3%. LB.1, identified for the first time in January 2024, reached an average abundance of 3% on May 2024. Similarly, KP.5, first identified in January 2024, reached 5% on June 2024. Interestingly, by the beginning of July 2024, KP.3* had increased to 81%, replacing most of the other lineages.

### 3.4. Phylogenetic Analysis

**Figure 5:**
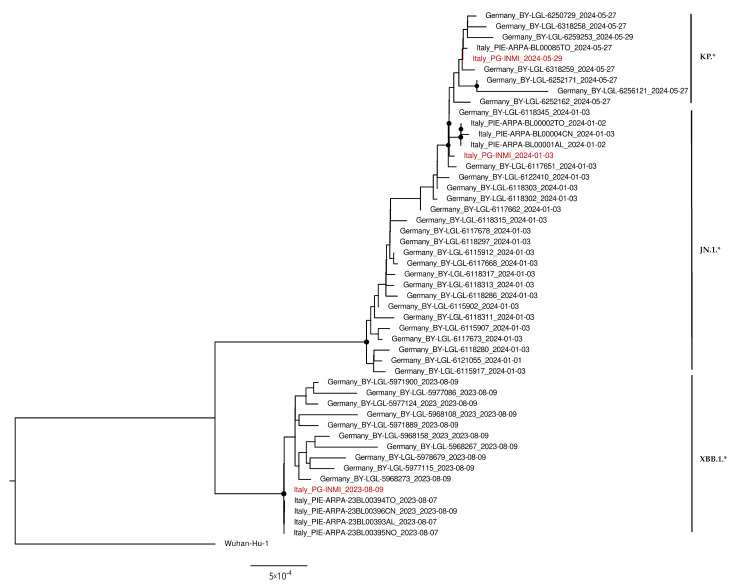
Phylogenetic tree of SARS-CoV-2 whole-genome sequences retrieved from wastewater samples representative of the evolution of the virus during the study period. Phylogenetic analysis was performed on wastewater samples collected on 9 August 2023, 3 January 2024, and 29 May 2024, and based on the maximum likelihood method applied to the whole-genome consensus sequences obtained (coded as Italy_PG-INMI_2023-08-09, Italy_PG-INMI_2024-01-03, and Italy_PG-INMI_2024-05-29, respectively, and reported in red in the phylogenetic tree), as well as other sequences retrieved from wastewater-based studies carried out during the same period in Italy and Europe (reported in black). Nodes supported with bootstrap values ≥80 are marked with black dots, while Pango lineages obtained by Nextclade (clades.nextstrain.org, accessed on 21 May 2025) are shown alongside the tree.

Three samples were selected for phylogenetic analysis at different timepoints, as representative of the evolution of the virus during the study period. In particular, samples collected on 9 August 2023, 3 January 2024, and 29 May 2024 were chosen because, as illustrated in previous paragraphs (Figure 2 and Figure 3), they were characterized by a prevalence of the three main SARS-CoV-2 lineages detected in the wastewater samples analyzed during the study, namely, XBB.*, JN.*, and KP.*, respectively. The phylogenetic tree reported in Figure 5 clearly indicates how the whole-genome sequences retrieved from our wastewater samples clustered closely with the lineages found in wastewater collected during the same period at both the national and European levels. As expected, the sample Italy_PG-INMI_2024-01-03 (identified as JN.1*) was distinguishable but clustered close to the Italy_PG-INMI_2024-05-29 sample (identified as KP.*), since they were both descendants of the BA.2.86 lineage. On the other hand, the sample Italy_PG-INMI_2023-08-09 (identified as XBB.1.*) appeared more phylogenetically distant.

### 3.5. Detection Timeline of SARS-CoV-2 Lineages in Wastewater Compared to Clinical Samples

**Figure 6:**
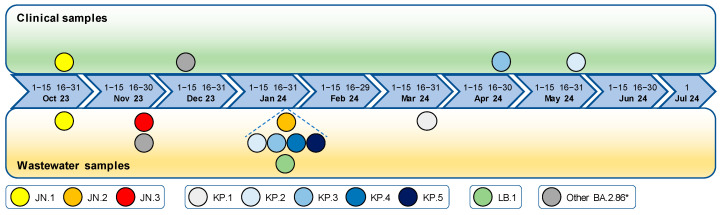
Timeline of BA.2.86 lineages’ identification by clinical genomic surveillance and by wastewater analysis: The period of the first detection of each BA.2.86 lineage (namely. JN.1 to JN.3, KP.1 to KP.5, LB.1. and other BA.2.86) is represented by the colored dots placed in the green upper panel and in the lower yellow panel for clinical and wastewater samples, respectively.

The main lineages detected in wastewater samples from August 2023 to July 2024 were compared with those identified in clinical samples collected during the same period from the local hospital and medical center (Table 1). Overall, the 236 clinical samples considered in this study allowed for the identification of 57 different lineages, while more than 400 were detected in wastewater samples. Most of the clinical samples (63.6%) were identified as one of the main Omicron XBB lineages that were also found in wastewater samples. Nevertheless, except for XBB.1.16 and EG.5, which appeared in both kinds of samples at virtually the same time, all other XBB lineages were identified earlier in wastewater samples (9 August 2023) than in clinical samples (18 August, 23 August, 11 September, and 15 September 2023, for XBB.1.5, XBB.2.3, XBB.1.9.2, and XBB.1.9.1, respectively). Interestingly, most lineages were detected for much longer (1 to 2 months) in wastewater samples than in clinical samples. Both approaches enabled the identification of the JN.1 subvariant from mid-October 2023. Nevertheless, while only one-third of clinical samples belonged to BA.2.86*, these lineages were found in the vast majority (87.8%) of wastewater samples. Figure 6 focuses on the identification timeline of the nine main circulating BA.2.86 subvariants: JN.1, JN.2, JN.3, KP.1, KP.2, KP.3, KP.4, KP.5, and LB.1 in clinical and wastewater samples. Most of these (JN.2, JN.3, KP.1, KP.4, KP.5, and LB.1) were never detected by clinical surveillance during the period of this study, while JN.1 was commonly found in both clinical and wastewater samples at the same time. In contrast, KP.2 and KP.3 were identified 3 to 4 months earlier in wastewater samples.

## 4. Discussion

The COVID-19 pandemic underscored the tight interdependence between human health and the health of domestic animals, wildlife, and the environment, a concept known as the One Health paradigm, which highlights how global anthropogenic changes represent key drivers of diseases’ emergence and spread. Under this One Health framework, surveillance of wastewater pathogens can integrate traditional disease-based surveillance to allow for timely detection and response to human and animal health threats, as well as contributing to the preparedness for future pandemics [55,56]. Wastewater surveillance was widely used during the COVID-19 pandemic to monitor epidemic trends, providing a non-invasive and comprehensive approach to capturing transmission at the community level. By capturing pooled genetic material from entire communities, this approach provides an unbiased and comprehensive overview of viral circulation, addressing key limitations of traditional clinical surveillance. Unlike clinical testing, which primarily focuses on symptomatic individuals or those actively seeking medical attention, wastewater analysis includes data from asymptomatic and mildly symptomatic individuals. Furthermore, the non-invasive and anonymous nature of wastewater monitoring ensures equitable data collection, bypassing barriers related to healthcare access or individual participation, making it a valuable tool for public health surveillance, particularly in under-resourced settings or regions with limited testing infrastructure [57,58,59]. While the initial focus was on detecting the presence of SARS-CoV-2 and estimating the prevalence of infection, the approach evolved to include sequencing of viral RNA as the virus began to mutate and generate numerous different lineages. This dual focus allowed researchers to simultaneously monitor overall viral circulation and identify the emergence and spread of specific variants of concern (VOCs) and variants of interest (VOIs), providing a more comprehensive understanding of viral evolution and community-level infection dynamics. By the end of 2021, the Omicron variant (B.1.1.529) and its subvariants, including BA.1, BA.2, and later BA.4 and BA.5, replaced Delta as the dominant variant. Omicron was notable for its extensive mutations in the spike protein, leading to significant immune escape while maintaining relatively low severity in most cases. In 2023, Omicron continued to diversify, with subvariants such as XBB.1.5* and XBB.2.3* causing waves of infections worldwide. In late 2023, BA.2.86* and its descendants, including JN.1*, began to emerge, followed by KP.1*, KP.2*, and KP.3* in 2024, confirming the virus’s continuing adaptation. The aims of this study were (1) to identify SARS-CoV-2 lineages circulating in the city of Perugia over a one-year period (August 2023–July 2024), with a particular focus on BA.2.86*, by wastewater analysis; and (2) to compare the data with those obtained by clinical surveillance in the same city and during the same period. In particular, we wanted to determine whether wastewater surveillance, as demonstrated in previous research, could provide broader insights by capturing viral shedding from both symptomatic and asymptomatic individuals, especially when the infection prevalence is low and, consequently, individual molecular testing is limited.

During the study period, a continuous shift in the prevalence of SARS-CoV-2 lineages over time was observed in sewage samples, reflecting the dynamic evolution of the virus. At the beginning of the study (August–October, 2023), Omicron XBB subvariants, mainly EG.5, were dominant. However, since mid-October 2023, XBB* was gradually replaced by BA.2.86*. In particular, JN.* (mainly JN.1) was the predominant BA.2.86* subvariant until the emergence of KP.*, primarily represented by KP.2 and KP.3. These observations were also evident in the phylogenetic tree, where the viral whole-genome sequences retrieved from the samples collected on August 9 2023, January 3 2024, and May 29 2024 (characterized by a prevalence of XBB.*, JN.*, and KP.*, respectively) were clearly phylogenetically distinguishable from each other, with XBB.1.* appearing more distant. These data mirrored those collected during the same period from clinical surveillance in Italy, reporting a predominance of XBB* until the emergence of BA.2.86* in late 2023. Moreover, the sequencing results obtained from the analyzed sewage confirmed the BA.2.86 circulation starting from mid-October, as already observed in Italy by RT-dPCR-based wastewater analysis [34]. Other surveys carried out in EU and non-EU countries detected the BA.2.86* lineages in wastewater at the beginning of its circulation within the population [38,60,61,62,63], highlighting the informative value of wastewater-based epidemiology (WBE) in the early detection of shifts in the prevalence of SARS-CoV-2 lineages.

By comparing data from wastewater and clinical surveillances, this study confirmed the ability of wastewater analysis to comprehensively capture the diversity of viral lineages circulating within the population, especially those with low prevalence. For instance, during this study, the subvariants JN.2, JN.3, KP.1, KP.4, KP.5, and LB.1 were detected in wastewater but not among clinical samples. Wastewater data also enabled the earlier detection of emerging lineages and allowed for their observation over an extended period compared to clinical epidemiology. This was particularly true for lineages characterized by a low clinical prevalence, such as KP.2 and KP.3, which were detected in wastewater several months prior to their detection in clinical cases. These discrepancies can be explained by the low representativeness of clinical data, which may be affected by the limited number of samples sequenced (236 over the one-year study period), typical of periods of low infection prevalence. In addition, the selection criteria for clinical samples (e.g., symptomatic individuals) may introduce bias, as asymptomatic and mildly symptomatic cases are less likely to be represented. These constraints strongly limit the ability of clinical data to fully represent the diversity of SARS-CoV-2 lineages circulating within the population. Indeed, the capacity of wastewater surveillance to detect rare lineages that are unlikely to appear in clinical datasets becomes crucial for understanding the genetic evolution of the virus and identifying potential threats before they become widespread. This early detection provides a critical time advantage for public health authorities, allowing for timely interventions, targeted epidemiological investigations, and informed resource allocation, especially on drugs, given that the clinical efficacy of anti-spike monoclonal antibodies can be inferred by immunobridging. This predictive capability highlights the utility of wastewater analysis as an early warning system for tracking potential outbreaks and anticipating shifts in variant prevalence [64].

Despite its strengths, this study presents known limitations of wastewater-based epidemiology, namely, the influence of factors such as population size, geographic coverage, sampling frequency, shedding rates, and daily wastewater flow on the representativeness of wastewater data. Additionally, technical issues as RNA stability in wastewater and the depth of sequencing could also impact the detection of smaller lineages. In particular, a limitation of this study is the reduced sampling frequency for August and September 2023, with only a single sample collected, in contrast with the weekly sampling in subsequent months, resulting in two months that may not fully represent the diversity and dynamics of circulating SARS-CoV-2 lineages.

In conclusion, this study demonstrates the complementary role of wastewater surveillance in filling gaps in clinical surveillance for monitoring the spread and evolution of SARS-CoV-2, especially during today’s post-pandemic low-incidence phase, as well as other viral pathogens. By combining RT-qPCR and whole-genome sequencing analyses of urban wastewater samples, the viral loads and the circulating lineages of SARS-CoV-2 in the city of Perugia were comprehensively assessed. In particular, two modest community waves were observed (September–December 2023 and May–July 2024), together with three successive Omicron subvariant phases (XBB.* dominance, BA.2.86/JN surge and KP.* takeover), indicating the continuing evolution of the virus during the study period. Furthermore, comparisons with data provided by clinical genomic surveillance showed how wastewater analysis could identify a much higher diversity of circulating viral lineages, allowing for an earlier and longer detection of some subvariants (including BA.2.86*). By providing early detection of lineages, comprehensive community-level data, and insights into viral evolution, wastewater monitoring has proven to be an indispensable tool for public health. Its scalability and adaptability also position it as a critical component of global pandemic preparedness, offering a robust framework for strengthening public health responses and building resilience against emerging infectious diseases.

## Figures and Tables

**Table 1:** SARS-CoV-2 lineages circulating in the study area from August 2023 to July 2024, as indicated by clinical genomic surveillance and by wastewater analysis. The main lineages detected in wastewater samples collected between 9 August 2023 and 1 July 2024 from the wastewater treatment plant of Pian della Genna (Perugia, Italy) (n = 42) were compared with those identified in clinical samples (respiratory tract) collected during the same period from the local hospital and medical center (n = 236). For each lineage, the number of samples, along with the relative frequency and the dates for the first and last detection, is reported for both clinical and wastewater samples.

		Clinical Samples			Wastewater Samples		
Lineage		Number (%)	First Detection	Last Detection	Number (%)	First Detection	Last Detection
XBB*		150 (63.6)	02 August 23	11 January 24	17 (41.5)	09 August 23	06 February 24
	XBB.1.16	13 (5.5)	02 August 23	15 December 23	5 (12.2)	09 August 23	30 October 23
	XBB.1.5	22 (9.3)	18 August 23	17 December 23	10 (24.4)	09 August 23	06 February 24
	XBB.2.3	5 (2.1)	23 August 23	07 October 23	6 (14.6)	09 August 23	08 November 23
	XBB.1.9.2	3 (1.3)	11 September 23	16 November 23	5 (12.2)	09 August 23	14 December 23
	XBB.1.9.1	9 (3.8)	15 September 23	04 December 23	14 (34.1)	09 August 23	06 February 24
	EG.5	97 (41.1)	07 August 23	11 January 24	16 (39.0)	09 August 23	06 February 24
	Other XBB*	1 (0.4)	26 October 23	26 October 23	12 (29.3)	09 August 23	11 January 24
BA.2.86*		78 (33.1)	16 October 23	27 June 24	36 (87.8)	18 October 23	01 July 24
	JN.1	71 (30.1)	16 October 23	27 June 24	33 (80.5)	18 October 23	01 July 24
	JN.2	0 (0.0)	n.d.	n.d.	1 (2.4)	24 January 24	24 January 24
	JN.3	0 (0.0)	n.d.	n.d.	4 (9.8)	29 November 23	27 March 24
	KP.1	0 (0.0)	n.d.	n.d.	3 (7.3)	20 March 24	29 May 24
	KP.2	1 (0.4)	31 May 24	31 May 24	7 (17.1)	24 January 24	19 June 24
	KP.3	3 (1.3)	27 April 24	14 May 24	10 (24.4)	24 January 24	01 July 24
	KP.4	0 (0.0)	n.d.	n.d.	4 (9.8)	24 January 24	12 June 24
	KP.5	0 (0.0)	n.d.	n.d.	2 (4.9)	24 January 24	04 June 24
	LB.1	0 (0.0)	n.d.	n.d.	7 (17.1)	24 January 24	08 May 24
	Other BA.2.86*	3 (1.3)	09 December 23	17 January 24	6 (14.6)	29 November 23	19 June 24
Other		8 (3.4)	10 September 23	21 May 24	21 (51.2)	09 September 23	27 March 24

## Data Availability

The data that support the findings of this study are available in a publicly accessible repository. FASTQ files were uploaded to the NCBI Sequence Read Archive (SRA) (accession number PRJNA1250441).
